# The Impact of Chitosan on the Chemical Composition of Wines Fermented with *Schizosaccharomyces* *pombe* and *Saccharomyces cerevisiae*

**DOI:** 10.3390/foods9101423

**Published:** 2020-10-09

**Authors:** Stefano Scansani, Doris Rauhut, Silvia Brezina, Heike Semmler, Santiago Benito

**Affiliations:** 1 Department of Microbiology and Biochemistry, Hochschule Geisenheim University (HGU), Von-Lade-Straße 1, 65366 Geisenheim, Germany; stefano.scansani@hs-gm.de (S.S.); doris.rauhut@hs-gm.de (D.R.); silvia.brezina@hs-gm.de (S.B.); heike.semmler@hs-gm.de (H.S.); 2 Department of Chemistry and Food Technology, Polytechnic University of Madrid, Ciudad Universitaria S/N, 28040 Madrid, Spain

**Keywords:** wine, aroma compounds, *Schizosaccharomyces*, chitosan, higher alcohols, esters, fatty acids, malic acid, *Saccharomyces*

## Abstract

This study investigates the influence of the antimicrobial agent chitosan on a selected *Schizosaccharomyces pombe* strain during the alcoholic fermentation of ultra-pasteurized grape juice with a high concentration of malic acid. It also studies a selected *Saccharomyces cerevisiae* strain as a control. The study examines several parameters relating to wine quality, including volatile and non-volatile compounds. The principal aim of the study is to test the influence of chitosan on the final chemical composition of the wine during alcoholic fermentation, and to compare the two studied fermentative yeasts between them. The results show that chitosan influences the final concentration of acetic acid, ethanol, glycerol, acetaldehyde, pyruvic acid, α-ketoglutarate, higher alcohols, acetate esters, ethyl esters, and fatty acids, depending on the yeast species.

## 1. Introduction

*Schizosaccharomyces pombe* is an interesting alternative to *Saccharomyces cerevisiae* for de-acidifying grape juices with a high malic acid content [1,2,3] or other fruit juices with similar acidity problems [4,5,6,7]. However, yeasts of the genus *Schizosaccharomyces* often produce undesirable effects during alcoholic fermentation, such as a high volatile acidity over 1 g L^−1^ [6,8] and high acetaldehyde production over 125 mg L^−1^ [4,9]. They also usually require longer fermentation times than *Sac. cerevisiae* [2,6]. 

In recent years, several authors have reported efforts to optimize the biotechnology of fermentation with *Sch. pombe* so as to avoid these undesirable effects. A combined inoculation with *Sac. cerevisiae* has enabled a reduction in the final concentration of the volatile acidity [2]. Several researchers have reported similar effects for combined fermentations with *Lachancea thermotolerans* [10,11,12,13] and *Torulaspora delbrueckii* [4]. Because of the high strain variability in parameters such as volatile acidity, which varies from 0.24 to 1.12 g L^−1^ [2], or acetaldehyde, which varies from 17 to 134.14 mg L^−1^ [2,4,9], it is possible to select small numbers of *Schizosaccharomyces* strains that do not exhibit these undesirable effects. Fed-batch fermentation technology with *Sch. pombe* allows for reducing acetic acid production during alcoholic fermentation by up to 100%, and leads to a 50% reduction in acetaldehyde production [14,15].

Other than deacidification, modern applications for *Sch. pombe* in fermentative industries include improvements in wine color [2], increased production of polysaccharides [9,16,17], and use as an aid to control food safety risks such as biogenic amines or ethyl carbamate [18,19].

The slower fermentation kinetics of *Sch. pombe* compared with those of *Sac. cerevisiae* often increase the risk of contamination at an industrial scale. The kinetics are even slower for fermentation at low temperatures [2], such as in the north of Europe. The antioxidant and antimicrobial agent sulfur dioxide usually solves the problems related to undesirable microbial contaminations during alcoholic fermentations involving *Sac. cerevisiae*. However, *Sch. pombe* is highly resistant to sulfur dioxide because its defense mechanisms is based on increasing the production of acetaldehyde, which combines with sulfur dioxide to inactivate the effect of its free form. Aditionally, it is also able to metabolize sulfur dioxide to sulfhydric acid reducing its effective concentration [2]. Thus, the use of antioxidant and antimicrobial agents other than sulfur dioxide could avoid the formation of these undesirable compounds during alcoholic fermentation with *Sch. pombe*.

Chitosan is a powerful antioxidant and antimicrobial [20,21,22,23,24] agent. It is becoming popular in winemaking because of a trend towards lowering the legal limit of sulfur dioxide in wines [18]. However, some studies report that chitosan has a lower antimicrobial effect compared with sulfur dioxide [25]. While there is evidence of the effect of chitosan on the fermentation kinetics of different yeast species [21], its influence on the final aroma composition remains unstudied. The present study explores the influence of chitosan on *Sch. pombe* alcoholic fermentation for the first time.

## 2. Experimental Section

### 2.1. Microorganisms

The *Sac. cerevisiae* strain Lalvin QA23^®^ (Lallemand, Montréal, QC, Canada) was the control sample for the vinification because of its rapid fermentation performance and high tolerance to low temperatures. The *Sch. pombe* strain V2 [10] possess a high rate of malic acid degradation and a low rate of acetic acid production.

### 2.2. Vinification

All of the fermentations used ultra-pasteurized (UHT) white grape juice (Jacoby GmbH, Auggen, Germany;). The amount of malic acid was adjusted by adding L-(−)-malic acid (Sigma Aldrich, Darmstadt, Germany), whereas the sugar level was adjusted by adding a mixture of D-glucose and D-fructose in an equal ratio (1:1; Merck KGaA, Darmstadt, Germany). The nitrogen level was adjusted by adding 0.4 g L^−1^ of Fermaid E Blanc (Danstar Ferment AG—Lallemand Inc., Fredericia, Denmark) and 0.4 g L^−1^ of OptiMUM white (Danstar Ferment AG—Lallemand Inc., Zug, Switzerland). The starting chemical parameters of the must after the adjustment were as follows: sugar 191.2 g L^−1^; pH 3.26; yeast-assimilable nitrogen content (YAN) 262 mg L^−1^ (Primary Amino Nitrogen (NOPA): 115 mg L^−1^ and ammonia: 146 mg L^−1^); tartaric acid, 2.33 g L^−1^; malic acid 6 g L^−1^; citric acid 0.15 g L^−1^; lactic acid <0.1 g L^−1^; and acetic acid < 0.1 g L^−1^. In two assays (SC_ch and SP_ch), 0.5 g L^−1^ of chitin-glucan and chitosan from *Aspergillus niger* origin were added following the manufacturer’s instructions (Bactiless™, Danstar Ferment AG—Lallemand Inc., Fredericia, Denmark) so as to evaluate the effect on the final wine chemical composition of the different species. 

Four assays were performed in triplicate according to a previously reported micro fermentation methodology [10] adapted to a 0.85 L scale, as follows: (1) inoculation of the must with *Sac. cerevisiae* QA23 (10^6^ cfu mL^−1^) alone (SC); (2) inoculation of the must with *Sac. cerevisiae* QA23 (10^6^ cfu mL^−1^) alone and 0.5 g L^−1^ of Bactiless™ (SC_ch); (3) inoculation of the must with *Sch. pombe* V2 (10^6^ cfu mL^−1^) alone (SP); and (4) inoculation of the must with *Sch. pombe* V2 (10^6^ cfu mL^−1^) alone and 0.5 g L^−1^ of Bactiless™ (SP_ch). The yeast inocula were prepared using 100 mL of sterilized must with 1 mL of yeast extract peptone dextrose (YEPD) liquid medium containing about 10^6^ cfu mL^−1^ (determined using a Thoma chamber hemocytometer). To reach this population, 100 μL of each yeast suspension was cultivated in 10 mL of YEPD at 25 °C for 24 h. This procedure was repeated three times before the final inoculation. The inoculations were performed in 250 mL sterile flasks sealed with a fermentation lock filled with an aqueous solution of 100 mg L^−1^ of total sulfur dioxide as potassium metabisulfite salt (Merck, Darmstadt, Germany), which allowed for the release of CO_2_ while avoiding microbial contamination. The temperature was maintained at 25 °C for 48 h. The development of the inocula was conducted without aeration, oxygen injection, or agitation.

All of the fermentation trials were carried out at 20 °C in a temperature-controlled room. The fermentation vessels were sealed with a fermentation lock filled with an aqueous solution of 100 mg L^−1^ of potassium metabisulfite (Merck, Darmstadt, Germany) that allowed for the release of CO_2_ while avoiding microbial contamination. The weight loss of the vessels during alcoholic fermentation was monitored using a scale model Kern FCB 12K1N (Kern and Sohn GmbH, Balingen, Germany). Once the weight loss remained constant for 48 h, the wines were racked and stabilized for 7 days at 4 °C, and the final product was bottled in 100 mL bottles. Potassium metabisulfite (Merck, Darmstadt, Germany) was then added to achieve a concentration of 80 mg L^−1^ total sulfur dioxide. The bottles were sealed with the headspace purged with nitrogen and were placed in a climate chamber at 4 °C.

### 2.3. Analytical Determinations

A pH 526 pH-meter equipped with a SennTix 81 electrode (WTW, Waldböckelheim, Germany) was used to measure the pH. Measurements of the non-volatile organic acids, ethanol, and residual sugars were performed by high-performance liquid chromatography (HPLC) according to Kanter et al., 2020 [26]. For the analyses of the esters, higher alcohols, and fatty acids, the method of the Department of Microbiology and Biochemistry of Hochschule Geisenheim University [27] was applied. The concentrations of acetaldehyde and pyruvate at the end of the fermentations were measured using specific enzymatic assays (K-ACHYD and K-PYRUV, Megazyme, Butzbach, Germany). The α-ketoglutarate was measured according to the Bergmeyer and Gawehn method [28].

### 2.4. Statistical Analysis

The statistical tests of this study were computed with R software (The R Foundation, v. 3.5.2 “Eggshell Igloo”), basing the analysis on the base R commands and the library agricolae [29]. The significance level was set at α = 0.05. The arithmetic means and standard deviations of the biological triplicates were calculated. The different treatments were analyzed by one-way ANOVA paired with a Ryan–Einot–Gabriel–Welsch F (REGWF) post-hoc test. When the ANOVA assumptions were not met, a non-parametric Van der Waerden test was run, coupled with a least significant difference (LSD) post-hoc test. A principal component analysis (PCA) was also performed on the dataset of the aroma active compounds.

## 3. Results and Discussion

### 3.1. Basic Chemical Parameters of the Wines

**Figure 1:**
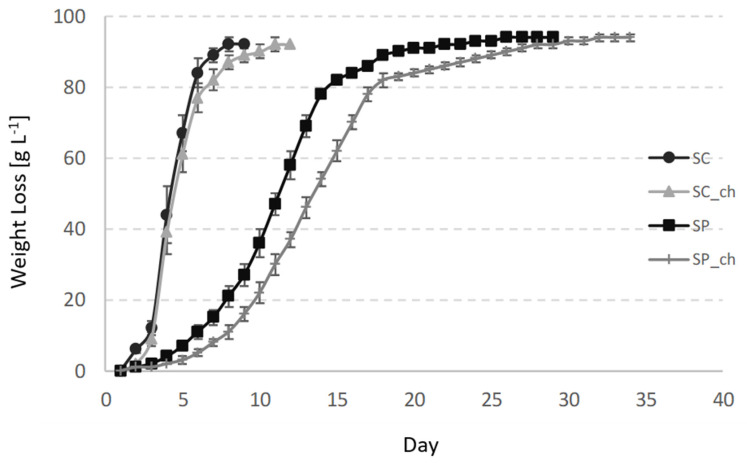
Fermentation kinetics of the variants measured gravimetrically by the total weight loss in the course of the fermentation. SC—*Sac. Cerevisiae*; SC_ch—*Sac. cerevisiae* fermenting with chitosan; SP—*Sch. Pombe*; SP_ch—*Sch. pombe* fermenting with chitosan. The values indicate the mean and standard deviations of the triplicates.

In all of the cases, *Sac. cerevisiae* and *Sch. pombe* completely consumed the sugars in the grape juice that fermented at 20 °C. The *Sac. cerevisiae* fermentations finished the alcoholic fermentations in 8 days (without chitosan) and 11 days (with chitosan), while *Sch. pombe* required 28 and 34 days, respectively, (Figure 1) because of the slower fermentation kinetics of that species at low temperatures [2]. A previous study reported that *Sch. pombe* requires a longer fermentation period than the *Sac. cerevisiae* controls under similar conditions, varying from 17 to 20 additional days [30]. Other studies reported shorter fermentations times for *Sch. pombe* fermentations, but at higher temperatures of between 25 and 30 °C [2]. In these studies, the *Sch. pombe* fermentations were often slower than the *Sac. cerevisiae* controls after about 2 to 4 days. The studies usually emphasized that *Sch. pombe* wines did not require malolactic fermentation any more, which significantly reduced the total red wine production time [13]. The *Sch. pombe* fermentations produced more ethanol than *Sac. cerevisiae,* by about 0.2 to 0.4% (*v/v*; Table 1). The unique capacity of the *Schizosaccharomcyes* genus to convert malic acid into ethanol and CO_2_ is the reason for this [2]. This effect is clearer in the fermentation of raw materials that contain higher levels of malic acid than grapes, such as kei-apple, which possess up to 45 g/L malic acid. Under these conditions, *Sch. pombe* produces alcoholic beverages with about 23% more ethanol than the *Sac. cerevisiae* control after fermenting all of the malic acid [6]. Some of the authors proposed the use of *Sch. pombe* in bioethanol production for its higher yield than *Sac. cerevisiae* [2]. The addition of chitosan did not influence the final ethanol concentration for *Sch. pombe* fermentations, and showed the same final concentration of 11.5% (*v/v*). *Sac. cerevisiae* produced 0.2% (*v/v*) less ethanol in the fermentation that contained chitosan.

The fermentations involving chitosan produced higher levels of glycerol. *Sch. pombe* produced 0.54 g L^−1^ more than *Sac. cerevisiae* made for the fermentations without chitosan. Some authors have suggested that *Sch. pombe* possesses a more developed glycerol–pyruvic pathway than *Sac. cerevisiae,* as beverages fermented by *Sch. pombe* often show higher levels of glycerol and pyruvic acid [13,31]. The *Sac. cerevisiae* fermentation enriched in chitosan produced 1.28 g L^−1^ more glycerol than the corresponding control. The *Sch. pombe* fermentation enriched in chitosan produced 0.8 g L^−1^ more glycerol than the control fermentation without chitosan.

Chitosan did not influence the final concentration of acetic acid for the *Sac. cerevisiae* fermentations, giving a final concentration of 0.35 g L^−1^. Previous studies reported that acetic acid has a slightly reduced final concentration (0.08 g/L lower) when chitosan performs as fining agent in wine [32]; however, that effect did not take place in this trial. Contrarily, chitosan increased the final acetic concentration of the *Sch. pombe* fermentations by about 0.1 g L^−1^. The final acetic acid concentrations in the *Sch. pombe* fermentation varied from 0.18 to 0.28 g L^−1^. All of the acetic acid concentrations were below the fault threshold of 0.8 g L^−1^ [29]. Although the *Schizosaccharomyces* genus can generate high concentrations of acetic acid over 1 g L^−1^ [2,6,8], modern strategies such as strain selection [2,10] sequential fermentations with other yeast [10,29], or fed-batch fermentation [14,15] enable the control of this undesirable effect [2].

*Sac. cerevisiae* fermentations degraded about 20% of the initial malic acid concentration. A slightly higher reduction of 0.1 g L^−1^ took place in the fermentation that contained chitosan. Chitosan can also adsorb significant amounts of malic acid—up to 20% when it performs as a fining agent [32]. The fermentations involving *Sch. pombe* completely degraded the initial 6 g L^−1^ of malic acid. *Sch. pombe* provided an efficient biological method to de-acidify acidic grape juices with malic acid contents over 6 g/L or other fruit juices with malic acid concentrations up to 45 g L^−1^ [6]. The degradation of malic acid influenced the final pH of wine, which varied from pH 3.22 for *Sac. cerevisiae* fermentations to pH 3.89 for *Sch. pombe* fermentations. Previous studies reported pH variations lower than about 0.1 when the malic acid initial content was below 1 g/L [2,13], and higher variations in a pH of about 0.9 for fruit with malic acid contents over 10 g/L [6].

The *Sac. cerevisiae* fermentations treated with chitosan showed a slightly lower final concentration of shikimic acid, by 0.48 mg/L compared with the *Sac. cerevisiae* control fermentation without chitosan, although no significant differences were observed (Table 1). Chitosan was able to adsorb shikimic acid similarly to that reported for malic acid [32]. Fermentations involving *Sch. pombe* showed the opposite effect, with an increase of about 1.1 mg/L for the fermentations treated with chitosan.

Previous studies reported a significant deacidification capacity for chitosan in fruit juices other than grape juice. The effect is because of chitosan interacting with organic acids [20]. In the present work, although slight differences in some organic acids took place, chitosan did not have statistically significant differences in the final pH.

**Figure 2:**
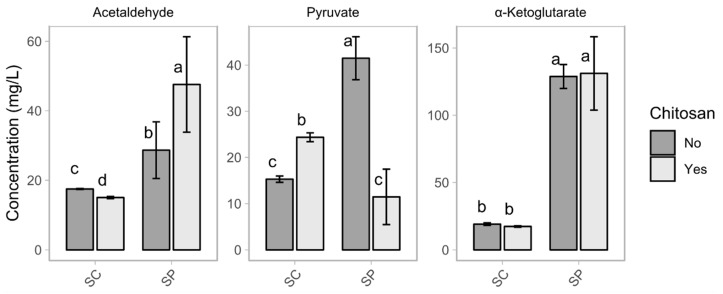
Plot of the concentrations of acetaldehyde, pyruvate, and α-ketoglutarate in the wines showing the variable yeast (SC and SP) and the variable chitosan (no and yes). The bars report the mean value and standard deviation. Within each plot, the same letters indicate a non-statistical difference between the mean values.

The *Sch. pombe* fermentations produced higher final concentrations of acetaldehyde than the *Sac. cerevisiae* fermentations (Figure 2). Chitosan influenced the production of acetaldehyde. In the case of *Sac. cerevisiae*, a slight decrease of 2.5 mg L^−1^ took place for the fermentations enriched with chitosan. In the case of *Sch. pombe,* the production of acetaldehyde increased from 28.6 to 47.6 mg L^−1^ in the presence of chitosan. Nevertheless, all of the acetaldehyde concentrations were below the fault aroma threshold of 125 mg L^−1^ [33,34]. Acetaldehyde production by members of the genus *Schizosaccharomyces* widely differ, depending on the strain, with reported values ranging from 16.46 mg L^−1^ to 134.14 mg L^−1^ [2,4,9]. A moderately high production of acetaldehyde below the fault threshold of 125 mg L^−1^ is of interest in the production of red wine, as it increases the content of one of the most stable anthocyanins, known as Vitisin B [2,13]. However, in white wine production, an excess of acetaldehyde may promote undesirable aromas, such as green apples or fresh-cut grass [13,33]. Using fed-batch technology during the alcoholic fermentation for white wine production reduces the production of acetaldehyde by *Sch. pombe* by 50% [14].

Chitosan influenced the production of pyruvic acid for the two species. The effect was an increase of about 59.4% in the case of *Sac. cerevisiae*, with concentrations that varied from 15.3 mg L^−1^ (SC) to 24.4 mg L^−1^ (SC_ch). However, the effect was a decrease of about 72.4% for fermentations by *Sch. pombe*, in which the final concentrations varied from 41.5 mg L^−1^ (SP) to 11.5 mg L^−1^ (SP_ch). *Sch. pombe* is the highest pyruvic acid producer among fermentative yeasts, able to reach concentrations four times higher than the *Sac. cerevisiae* controls [2,13,31].

Chitosan did not influence the production of α-ketoglutarate. The *Sch. pombe* fermentations produced final concentrations of α-ketoglutarate of about 128.8 mg L^−1^ (SP) and 131.1 mg L^−1^ (SP_ch), while the *Sac. cerevisiae* fermentations produced final concentrations of about 19.1 mg L^−1^ (SC) and 17.1 mg L^−1^ (SC_ch).

### 3.2. Volatile Compounds

Some non-conventional yeasts can improve wine aroma complexity because of their influence over higher alcohols, esters, and terpenes [35,36,37,38,39]. Previous studies reported that selected *Sch. pombe* strains produce lower concentrations of higher alcohols than the *Sac. cerevisiae* controls, varying from 25% to 77% depending on the studied higher alcohol [2,10]. Reducing the production of higher alcohols is a modern strategy to avoid masking desirable varietal aromas [33]. The present study observed a reduction of about 50% of total higher alcohols for *Sch. pombe* fermentations compared with the *Sac. cerevisiae* controls (Table 2). *Sch. pombe* produced lower isoamyl alcohol, 2-phenylethyl alcohol, and isobutanol than the *Sac. cerevisiae* control by 56.0%, 64.3%, and 26.7%, respectively. Chitosan did not significantly influence higher alcohol production for the *Sac. cerevisiae* fermentations. In the case of *Sch. pombe,* the chitosan fermentations produced significantly lower levels of isoamyl alcohol and 2-phenyl alcohol. Other studies reported that chitosan can remove significant amounts of 4-ethylphenol and 4-ethylguaiacol from red wine, varying from 7 to 25% depending on the dose [40].

*Schizosaccharomyces* fermentations produced a lower concentration of esters than those of *Sac. cerevisiae*, with the effect being especially noteworthy for the ethyl esters family where the observed reduction was 22% (Table 2). Previous studies reported reductions in ester production by *Sch. pombe* compared with the *Sac. cerevisiae* controls, with the effect varying from 15 to 35%, depending on the studied ester [2,10]. Chitosan stimulated the production of acetate esters such as isoamyl acetate and phenyl ethyl acetate by 44.6% and 37.2%, respectively, for *Sac. cerevisiae*. The opposite effect took place for *Sch. pombe,* with chitosan causing a 28.4% reduction in ethyl acetate, a 49.3% reduction in isoamyl acetate, a 28.2% reduction in 2-methylbutyl acetate, and a 45.9% reduction in phenyl acetate. It was not possible to quantify hexyl acetate in the *Sch. pombe* fermentation enriched with chitosan.

Chitosan did not influence the final concentration of ethyl esters for the fermentations with *Sac. cerevisiae*. Other studies reported reductions in some esters, such as methyl and ethyl octanoate, showing about a 30% reduction when chitosan performs as a fining agent [32]. In the case of *Sch. pombe*, the chitosan trials showed higher final concentrations in ethyl isobutyrate (47.8%) and ethyl propionate (27.39%), while they showed lower concentrations of ethyl butyrate (42.6% reduction), ethyl hexanoate (17.9% reduction), and ethyl decanoate (42.1% reduction).

*Sch. pombe* produced less fatty acids than *Sac. cerevisiae* for all of the fatty acids included in our study. The biggest differences took place for isovaleric, octanoic, and decanoic acids (with reductions of 26.0%, 34.9%, and 34.2%, respectively). Chitosan did not influence the final concentration of fatty acids in the case of *Sac. cerevisiae*, while some fatty acid concentrations decreased in the *Sch. pombe* fermentations, such as isovaleric acid (13.3% reduction), hexanoic acid (12.3% reduction), and decanoic acid (23.0% reduction).

Previous studies reported significant reductions (up to 70%) in terpene concentrations when chitosan performed as a fining agent in a terpenic grape variety [32]. Chitosan rarely affected the other aroma compounds. In the present study, although the wines did not show a high content in the terpenes, a statistically significant increase of 1.2 µg/L terpenes took place for the *Sch. pombe* fermentations containing chitosan.

**Figure 3:**
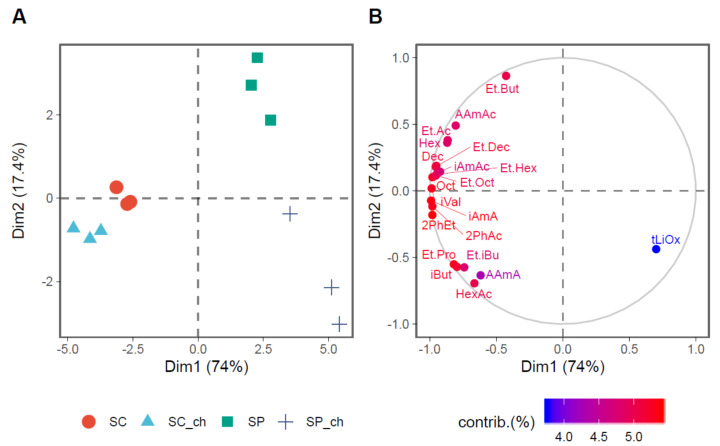
Principal component analysis of the microbial metabolic contribution to the aroma of the wines. (**A**) The plot of the fermentation treatments in the two principal dimensions (Dim1 and Dim2), which indicates 91.7% of the variance in the data. (**B**) The plot of the first 15 variables with the highest contribution on the explained variance between treatments in the two dimensions is presented in a continuous scale of colors that show the lowest contribution (blue) to the highest (red). Abbreviations: Ethyl acetate (Et.Ac), Isobutanol (iBut), Ethyl propionate (Et.Pro), Isoamyl alcohol (iAmA), Active amyl alcohol (AAmA), Ethyl isobutyrate (Et.iBu), Ethyl butyrate (Et.But), Isovaleric acid (iVal), Isoamyl acetate (iAmAc), Active amyl acetate (AAmAc), Hexanoic acid (Hex), Ethyl hexanoate (Et.Hex), Hexyl acetate (HexAc), trans-Linalool oxide (tLiOx), 2-Phenylethanol (2PhEt), Octanoic acid (Oct), Ethyl octanoate (Et.Oct), 2-Phenylethyl acetate (2PhAc), Decanoic acid (Dec), and Ethyl decanoate (Et.Dec).

The PCA in Figure 3 shows the lower overall contribution of *Sch. pombe* to the final fermentation aroma compared with *Sac. cerevisiae*. *Sac. cerevisiae* differs from *Sch. pombe* because of the increased production of aroma-active compounds. The PCA also highlights the different behavior of yeasts in the presence of chitosan. Chitosan shows a slight effect on the composition of the *Sac. cerevisiae* aroma compounds, which does not affect the final fermentation aroma because the variants are closely grouped. However, in the case of *Sch. pombe*, the variant fermented with chitosan separated from the one without because of a lower aroma production.

## 4. Conclusions

Chitosan is an important tool for controlling oxidation and undesirable microbial development during fermentation and wine ageing. Our findings show that chitosan influences the final chemical composition of wine fermented with *Sac. cerevisiae* and *Sch. pombe*, including the contents of acetic acid, ethanol, glycerol, acetaldehyde, pyruvic acid, α-ketoglutarate, higher alcohols, acetate esters, ethyl esters, and volatile fatty acids. The results of the study also show that *Sch. pombe* produces higher concentrations of glycerol, acetaldehyde, pyruvate, and α-ketoglutarate than the *Sac. cerevisiae* control. Contrarily, the selected *Sch. pombe* strain produced lower levels of acetic acid, malic acid, higher alcohols, ethyl esters, and fatty acids.

## Figures and Tables

**Table 1:** Final analysis of the basic fermentative parameters—values from wines fermented with *Sac. cerevisiae* (SC), *Sac. cerevisiae* fermenting with chitosan (SC_ch), *Sch. pombe* (SP), and *Sch. pombe* fermenting with chitosan (SP_ch).

Parameter	SC	SC_ch	SP	SP_ch
Ethanol [% *v*/*v*]	11.26 ± 0.13 ^b^	11.08 ± 0.01 ^b^	11.52 ± 0.06 ^a^	11.52 ± 0.09 ^a^
Residual Sugars [g L^−1^]	<2	<2	<2	<2
Glucose [g L^−1^]	n.q.	n.q.	n.q.	n.q.
Fructose [g L^−1^]	n.q.	n.q.	n.q.	n.q.
Glycerol [g L^−1^]	7.20 ± 0.03 ^c^	8.48 ± 0.14 ^a^	7.74 ± 0.17 ^b^	8.54 ± 0.41 ^a^
Tartaric acid [g L^−1^]	1.24 ±0.02 ^a^	1.23 ±0.02 ^a^	1.10 ± 0.01 ^b^	1.28 ± 0.06 ^a^
L-Malic acid [g L^−1^]	4.75 ± 0.01 ^a^	4.65 ± 0.03 ^b^	n.q.	n.q.
Shikimic acid [mg L^−1^]	8.32 ± 0.09 ^b^	7.84 ± 0.19 ^b^	8.27 ± 0.05 ^b^	9.40 ± 0.63 ^a^
L-Lactic acid [g L^−1^]	0.18 ± 0.01 ^b^	0.20 ± 0.00 ^a,b^	0.21 ± 0.00 ^a^	0.20 ± 0.02 ^a,b^
Acetic acid [g L^−1^]	0.35 ± 0.00 ^a^	0.35 ± 0.01 ^a^	0.18 ± 0.02 ^c^	0.28 ± 0.02 ^b^
Citric acid [g L^−1^]	0.16 ± 0.01 ^a^	0.16 ± 0.01 ^a^	0.17 ± 0.02 ^a^	0.16 ± 0.03 ^a^
pH	3.26 ± 0.02 ^b^	3.22 ± 0.02 ^b^	3.89 ± 0.01 ^a^	3.89 ± 0.02 ^a^

The values indicate the mean and standard deviations of the fermentation triplicates; mean values in the same row with the same superscript letter are not significantly different from each other (*p* < 0.05); n.q.—not quantifiable.

**Table 2:** Volatile compounds measured after the fermentation of *Sac. cerevisiae* (SC), *Sac. cerevisiae* fermenting with chitosan (SC_ch), *Sch. pombe* (SP), and *Sch. pombe* fermenting with chitosan (SP_ch).

Volatile Compound	SC	SC_ch	SP	SP_ch
**Acetate Esters**				
Ethyl Acetate [mg L^−1^]	169.8 ± 9.6 ^b^	187.0 ± 1.2 ^a^	163.6 ± 5.3 ^b^	117.2 ± 5.5 ^c^
Isoamyl acetate [µg L^−1^]	7813.4 ± 374.3 ^b^	11,301.5 ± 1197.0 ^a^	6350.6 ± 286.3 ^b^	3217.3 ± 1643.5 ^c^
2-Methyl butyl acetate [µg L^−1^]	261.1 ± 10.7 ^a^	280.3 ± 9.1 ^a^	254.0 ± 9.6 ^a^	182.5 ± 43.4 ^b^
Hexyl acetate [µg L^−1^]	88.1 ± 4.9 ^a^	98.5 ± 10.4 ^a^	31.0 ± 2.4 ^b^	n.q.
2-Phenyl ethyl acetate [µg L^−1^]	330.5 ± 4.0 ^b^	453.4 ± 36.4 ^a^	123.1 ± 11.6 ^c^	66.6 ± 30.3 ^d^
Ʃ Acetates [µg L^−1^]	178,293.1	199,133.7	170,358.7	15,186.4
**Ethyl Esters**				
Ethyl propionate [µg L^−1^]	170.1 ± 4.5 ^b^	187.4 ± 4.5 ^a^	93.6 ± 12.1 ^d^	128.9 ± 5.1 ^c^
Ethyl butyrate [µg L^−1^]	552.6 ± 12.1 ^a^	562.1 ± 53.7 ^a^	657.8 ± 16.6 ^a^	377.7 ± 102.5 ^b^
Ethyl isobutyrate [µg L^−1^]	39.7 ± 3.4 ^a^	41.4 ± 3.2 ^a^	20.3 ± 1.6 ^c^	30.1 ± 5.9 ^b^
Ethyl hexanoate [µg L^−1^]	907.1 ± 16.0 ^a^^,^^b^	1006.2 ± 103.1 ^a^	777.0 ± 49.7 ^b^^,^^c^	637.7 ± 64.7 ^c^
Ethyl octanoate [µg L^−1^]	1354.4 ± 26.6 ^a^	1293.8 ± 48.9 ^a^	917.6 ± 68.4 ^b^	749.2 ± 196.5 ^b^
Ethyl decanoate [µg L^−1^]	464.1 ± 4.7 ^a^	430.6 ± 28.6 ^a^	281.3 ± 26.0 ^b^	162.8 ± 59.5 ^c^
Ʃ Ethyl esters [µg L^−1^]	3488.0	3521.5	2747.6	2086.4
**Higher Alcohols**				
Isobutanol [mg L^−1^]	28.8 ± 0.5 ^b^	32.0 ± 1.3 ^a^	20.6 ± 1.2 ^d^	24.0 ± 2.5 ^c^
Isoamyl alcohol [mg L^−1^]	243.7 ± 7.8 ^a^	255.1 ± 7.4 ^a^	126.2 ± 5,7 ^b^	93.3 ± 7.5 ^c^
Active amyl alcohol [mg L^−1^]	22.4 ± 0.2 ^a^^,^^b^	26.3 ± 0.4 ^a^	19.3 ± 2.2 ^b^	21.6 ± 2.3 ^b^
Hexanol [µg L^−1^]	n.q.	n.q.	n.q.	n.q.
2-Phenyl−ethanol [mg L^−1^]	18.6 ± 0.9 ^b^	22.0 ± 0.5 ^a^	7.9 ± 0.4 ^c^	6.6 ± 0.4 ^d^
Ʃ Higher alcohols [mg L^−1^]	313.5	335.4	174	145.5
**Fatty Acids**				
Isovaleric acid [µg L^−1^]	2526.2 ± 103.2 ^a^	2620.7 ± 53.0 ^a^	2040.2 ± 7.4 ^b^	1768.6 ± 18.3 ^c^
Hexanoic acid [mg L^−1^]	12.9 ± 0.7 ^a^	12.9 ± 0.4 ^a^	12.1 ± 0.6 ^a^	10.6 ± 0.5 ^b^
Octanoic acid [mg L^−1^]	10.8 ± 0.5 ^a^	11.0 ± 0.5 ^a^	7.9 ± 0.6 ^b^	6.3 ± 1.0 ^b^
Decanoic acid [mg L^−1^]	3.1 ± 0.1 ^a^	3.0 ± 0.2 ^a^	2.3 ± 0.2 ^b^	1.8 ± 0.4 ^c^
Ʃ Fatty acids [mg L^−1^]	29.3	29.5	24.3	20.5
**Monoterpenoids and C_13_-norisoprenoids**				
Linalool oxide-1 [µg L^−1^]	5.4 ± 0.5 ^b^	5.9 ± 0.6 ^b^	6.0 ± 0.3 ^b^	7.2 ± 0.2 ^a^

The values indicate the mean and standard deviation of the fermentation triplicates; mean values in the same row with the same letter are not significantly different from each other (*p* < 0.05); n.q.—not quantifiable.
